# Supplementary material for: Resolving the full spectrum of human genome variation using Linked-Reads
Source: Genome Res. 2019 Apr;29(4):635–45. doi: 10.1101/gr.234443.118 (PMC6442396; doi:10.1101/gr.234443.118)
Supplement: Supplemental Material [file supp_gr.234443.118_Supplemental_longranger-2.2.zip › longranger-2.2.tar.bz2!/lr2.2-public/lib/stan/cmdstan/stan/doc/api/html/json__data_8hpp_source.html]

Stan: src/stan/io/json/json\_data.hpp Source File


|  |  |
| --- | --- |
|  | Stan  2.5.0  probability, sampling & optimization |


- Main Page
- Related Pages
- Namespaces
- Classes
- Files

- File List
- File Members

All Classes Namespaces Files Functions Variables Typedefs Enumerations Enumerator Friends Macros Pages

- src
- stan
- io
- json

json\_data.hpp

Go to the documentation of this file.

1 #ifndef STAN\_\_IO\_\_JSON\_\_JSON\_DATA\_HPP

2 #define STAN\_\_IO\_\_JSON\_\_JSON\_DATA\_HPP

3

4 #include <cctype>

5 #include <iostream>

6 #include <limits>

7 #include <map>

8 #include <sstream>

9 #include <string>

10 #include <vector>

11 #include <boost/throw\_exception.hpp>

12 #include <boost/lexical\_cast.hpp>

13 #include <stan/math/matrix.hpp>

14 #include <stan/io/var\_context.hpp>

15 #include <stan/io/json/json\_error.hpp>

16 #include <stan/io/json/json\_parser.hpp>

17 #include <stan/io/json/json\_data\_handler.hpp>

18

19

20 namespace stan {

21

22  namespace json {

23

46  class json\_data : public stan::io::var\_context {

47  private:

48  vars\_map\_r vars\_r\_;

49  vars\_map\_i vars\_i\_;

50

51  std::vector<double> const empty\_vec\_r\_;

52  std::vector<int> const empty\_vec\_i\_;

53  std::vector<size\_t> const empty\_vec\_ui\_;

54

64  bool contains\_r\_only(const std::string& name) const {

65  return vars\_r\_.find(name) != vars\_r\_.end();

66  }

67

68  public:

69

78  json\_data(std::istream& in) : vars\_r\_(), vars\_i\_() {

79  json\_data\_handler handler(vars\_r\_, vars\_i\_);

80  stan::json::parse(in, handler);

81  }

82

91  bool contains\_r(const std::string& name) const {

92  return contains\_r\_only(name) || contains\_i(name);

93  }

94

103  bool contains\_i(const std::string& name) const {

104  return vars\_i\_.find(name) != vars\_i\_.end();

105  }

106

114  std::vector<double> vals\_r(const std::string& name) const {

115  if (contains\_r\_only(name)) {

116  return (vars\_r\_.find(name)->second).first;

117  } else if (contains\_i(name)) {

118  std::vector<int> vec\_int = (vars\_i\_.find(name)->second).first;

119  std::vector<double> vec\_r(vec\_int.size());

120  for (size\_t ii = 0; ii < vec\_int.size(); ii++) {

121  vec\_r[ii] = vec\_int[ii];

122  }

123  return vec\_r;

124  }

125  return empty\_vec\_r\_;

126  }

127

135  std::vector<size\_t> dims\_r(const std::string& name) const {

136  if (contains\_r\_only(name)) {

137  return (vars\_r\_.find(name)->second).second;

138  } else if (contains\_i(name)) {

139  return (vars\_i\_.find(name)->second).second;

140  }

141  return empty\_vec\_ui\_;

142  }

143

151  std::vector<int> vals\_i(const std::string& name) const {

152  if (contains\_i(name)) {

153  return (vars\_i\_.find(name)->second).first;

154  }

155  return empty\_vec\_i\_;

156  }

157

165  std::vector<size\_t> dims\_i(const std::string& name) const {

166  if (contains\_i(name)) {

167  return (vars\_i\_.find(name)->second).second;

168  }

169  return empty\_vec\_ui\_;

170  }

171

178  virtual void names\_r(std::vector<std::string>& names) const {

179  names.resize(0);

180  for (vars\_map\_r::const\_iterator it = vars\_r\_.begin();

181  it != vars\_r\_.end(); ++it)

182  names.push\_back((\*it).first);

183  }

184

191  virtual void names\_i(std::vector<std::string>& names) const {

192  names.resize(0);

193  for (vars\_map\_i::const\_iterator it = vars\_i\_.begin();

194  it != vars\_i\_.end(); ++it)

195  names.push\_back((\*it).first);

196  }

197

205  bool remove(const std::string& name) {

206  return (vars\_i\_.erase(name) > 0)

207  || (vars\_r\_.erase(name) > 0);

208  }

209

210  };

211

212  }

213

214 }

215 #endif

stan::json::json\_data::vals\_r

std::vector< double > vals\_r(const std::string &name) const

Return the double values for the variable with the specified name or null.

**Definition:** json\_data.hpp:114

stan::json::json\_data::contains\_r

bool contains\_r(const std::string &name) const

Return true if this json\_data contains the specified variable name.

**Definition:** json\_data.hpp:91

json\_parser.hpp

stan::json::json\_data::vals\_i

std::vector< int > vals\_i(const std::string &name) const

Return the integer values for the variable with the specified name.

**Definition:** json\_data.hpp:151

stan::json::json\_data\_handler

A json\_data\_handler is an implementation of a json\_handler that restricts the allowed JSON text a set...

**Definition:** json\_data\_handler.hpp:54

stan::json::json\_data::contains\_i

bool contains\_i(const std::string &name) const

Return true if this json\_data contains an integer valued array with the specified name...

**Definition:** json\_data.hpp:103

json\_error.hpp

stan::json::json\_data::json\_data

json\_data(std::istream &in)

Construct a json\_data object from the specified input stream.

**Definition:** json\_data.hpp:78

var\_context.hpp

stan::io::var\_context

A var\_reader reads array variables of integer and floating point type by name and dimension...

**Definition:** var\_context.hpp:29

stan::json::json\_data::names\_i

virtual void names\_i(std::vector< std::string > &names) const

Return a list of the names of the integer variables in the json\_data.

**Definition:** json\_data.hpp:191

stan::json::json\_data::names\_r

virtual void names\_r(std::vector< std::string > &names) const

Return a list of the names of the floating point variables in the json\_data.

**Definition:** json\_data.hpp:178

stan::json::json\_data::dims\_i

std::vector< size\_t > dims\_i(const std::string &name) const

Return the dimensions for the integer variable with the specified name.

**Definition:** json\_data.hpp:165

stan::json::parse

void parse(std::istream &in, Handler &handler)

Parse the JSON text represented by the specified input stream, sending events to the specified handle...

**Definition:** json\_parser.hpp:429

json\_data\_handler.hpp

stan::json::json\_data::dims\_r

std::vector< size\_t > dims\_r(const std::string &name) const

Return the dimensions for the variable with the specified name.

**Definition:** json\_data.hpp:135

stan::json::json\_data

A json\_data is a var\_context object that represents a set of named values which are typed to either d...

**Definition:** json\_data.hpp:46

matrix.hpp


---

[ Stan Home Page ]

*© 2011–2014,
Stan Development Team.*
